# Data on the in-vitro digestibility of acid gels prepared from brewers’ spent grain protein isolates

**DOI:** 10.1016/j.dib.2021.107160

**Published:** 2021-05-21

**Authors:** Thierry Hellebois, Claire Gaiani, Cédric Paris, Sébastien Planchon, Jenny Renaut, Christos Soukoulis

**Affiliations:** a Environmental Research and Innovation (ERIN) Department, Luxembourg Institute of Science and Technology (LIST), 5 avenue des Hauts Fourneaux, L-4362, Esch-sur-Alzette, LUXEMBOURG; b Université de Lorraine, LIBio, F-54000 Nancy, FRANCE

**Keywords:** Plant protein, Hydrogel, Protein digestibility, Thermal treatment, In-vitro digestion

## Abstract

Brewers’ Spent Grain (BSG) is the primary waste of the beer brewing process, which comprises a plethora of nutritionally appealing ingredients such as proteins, dietary fibres, essential lipids and micronutrients. In our previous study [1], the acid-induced gelation capacity of BSG protein isolate as influenced by the thermal pre-treatment severity was systematically investigated. In the present work, we aimed at providing a dataset outlining the gastrointestinal fate of the acid gels under simulating pre-absorptive digestion conditions adopting the INFOGEST static in-vitro digestion protocol. Protein hydrogel digestibility was assessed by quantification of the total soluble nitrogen content in the initial acid gels as well as the obtained gastric and small intestine chymes. The extent of proteolysis occurring in the oral, gastric and intestinal phases was investigated by SDS-PAGE and the molecular weight distribution of the proteins in the obtained gastric chymes and intestinal digesta was determined by image analysis. The dataset can be deployed to assist food scientists in the design and development of alternative protein-based food and food supplement products adopting the “waste-to-fork” concept.

## Specifications Table

**Table:** 

Subject	Agricultural and Biological Sciences
Specific subject area	Food Sciences
Type of data	Figures, table, images
How data were acquired	The acid gels’ gastrointestinal fate was assessed by SDS-PAGE (Bio-Rad Criterion™) and data acquisition was registered by means of laser scanner (Typhoon FLA 9500 Imager). Image analysis of the SDS-PAGE gel was conducted using the Image J image processing program. The in-vitro digestibility was monitored by determining the soluble nitrogen index using the Kjeldahl method (Kjeldahl titrator, Gerhardt, Germany). The amino acid composition was quantified by LC-MS (LTQ^XL^, ThermoFisher Scientific, San Jose, CA, USA) after acid hydrolysis.
Data format	Raw and analysed
Parameters for data collection	Brewers’ spent grain protein isolate (BSG-PI) dispersions (5% wt) were subjected into five thermal pre-treatment conditions: unheated (native), mild to high (72, 85 or 92 °C for 20 min) and severe heat treatment (autoclaving, 121 °C for 15 min). The acid gels were produced via δ-glucono-lactone (1.2% wt) mediated acidification of the BSG-PI dispersions and then, they were subjected to in-vitro digestion conditions according to the INFOGEST protocol specifications.
Description of data collection	Acid gels underwent simulated pre-absorptive digestion adopting the INFOGEST static in-vitro digestion model. Aliquots of the oral (t_I_ = 2 min), start (t_GS_ = 0 min) and end (t_GE_ = 60 min) gastric and end intestinal (t_INT_ = 120 min) phases were assessed by SDS-PAGE and soluble nitrogen content. BSG-PI amino acid composition of the native powder isolate was also determined.
Data source location	Data were collected at the Luxembourg Institute of Science and Technology, Belvaux, Luxembourg. Latitude and longitude for collected data: 49.50623 N, 5.94381 E.
Data accessibility	Repository name: Mendeley data
	Data identification number: 10.17632/3jbc338f58.1
	Direct URL to data: http://dx.doi.org/10.17632/3jbc338f58.1
Related research article	T. Hellebois, C. Gaiani, S. Planchon, J. Renaut, and C. Soukoulis, Impact of heat treatment on the acid induced gelation of brewers’ spent grain protein isolates, Food Hydrocolloids. DOI:10.1016/j.foodhyd.2020.106531

## Value of the Data

• Experimental evidence of the performance of acid gels made from thermally treated brewers’ spent grain protein isolate under in-vitro digestion conditions is provided for the first time.
• The dataset article can be used by R&D scientists working in the design of alternative protein-based food products and/or food supplements adopting the “waste-to-fork” concept.
• The dataset provides a technical roadmap to determine the gastrointestinal fate of alternative protein ingredients and their derivatives thereof.
• Notwithstanding the heat treatment conditions, all acid gels exerted a quite restricted protein digestibility (less than 10 − 15%) under in-vitro gastric conditions.
• BSG-PI acid gels achieved very high digestibility rates (up to 93%) under simulated duodenal conditions.

## 1 Data Description

The present data article aimed at providing a preliminary outlook on the digestibility of BSG-PI based acid gels under standardized in-vitro digestion conditions (INFOGEST model). It is envisaged that the data article can assist food scientists, working in the field of functional food innovation, to identify the feasibility of BSG-PI acid gels for the development of alternative foods or food supplements. For emphasis purposes, the data presents only analyzed data whereas all the raw data used are stored in the online public repository linked above.

**Fig. 1:**
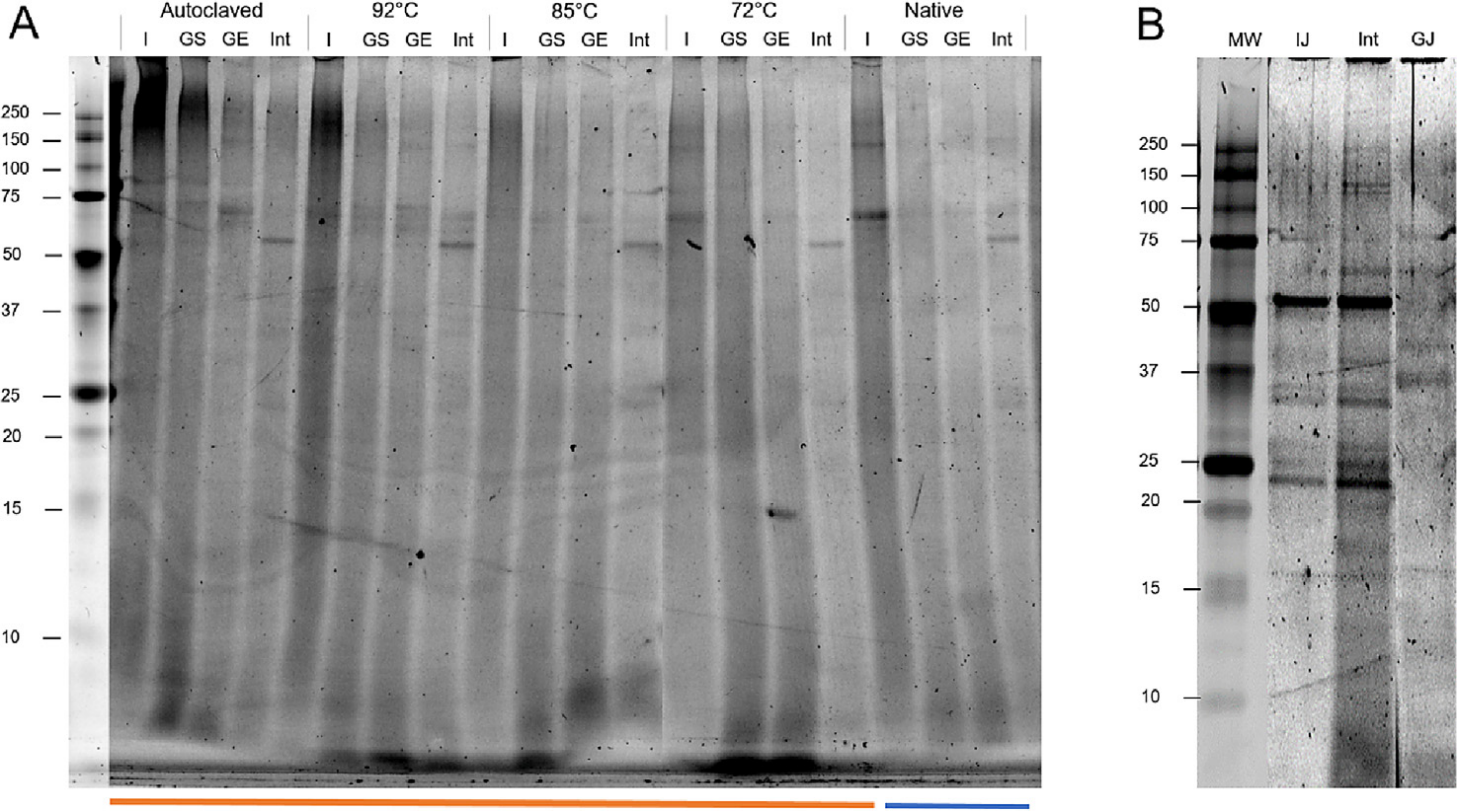
A: Changes in the molecular weight distribution occurring throughout in-vitro digestion (INFOGEST protocol) of BSG-PI acid gels. *I* = oral bolus (t_I_ = 2 min), GS: gastric chyme (t_GS_ = 0 min), GE = gastric chyme (t_GE_ = 60 min), Int = intestinal chyme (t_INT_ = 120 min). B: SDS-PAGE gels, the pure gastric (GJ) and intestinal juices (IJ) were run together with a reference BSG-PI digesta system (Int).

Fig 1A. displays an analyzed image of “SDS-PAGE Gel 3” from the three replicates of the repository in the original format (SDS-PAGE gel 1–3). Fig 1B shows the molecular weight distribution profile of the model in-vitro digestive fluids (without the addition of food matrix) and generated by the analysis of the “SDS-PAGE Gel 4” image. For better understanding and reuse of the raw images, a complete description of the lanes is given in the sheet “SDS-PAGE Lanes information” of the annexed Excel file “digestibility”. As seen in Fig. 1A, a decrease in the intensity of the high M_W_ bands (> 37 kDa) followed by a mutual increase in the intensity of the low M_W_ bands (< 10–15 kDa) was observed. Increasing the severity of the heat pre-treatment of the BSG-PI dispersions was associated with a progressive arise in the signal intensity of the high molecular weight bands (> 75 kDa). That is primarily attributed to the formation of glutelin and hordein monomers/oligomers aggregates during the heating process [1,2]. In the intestinal chymes, all protein bands exhibited a substantially low signal intensity suggesting a high degree of proteolytic activity. No clear bands were identified in all systems (apart from the lanes corresponding to the digestive enzymes such as pepsin and pancreatin, Fig. 1B); instead, a quite broad protein signal intensity pattern was observed, most probably due to extensive proteolytic attack of the proteinaceous matter of the barley grains during the malting and mashing steps [3].

**Fig. 2:**
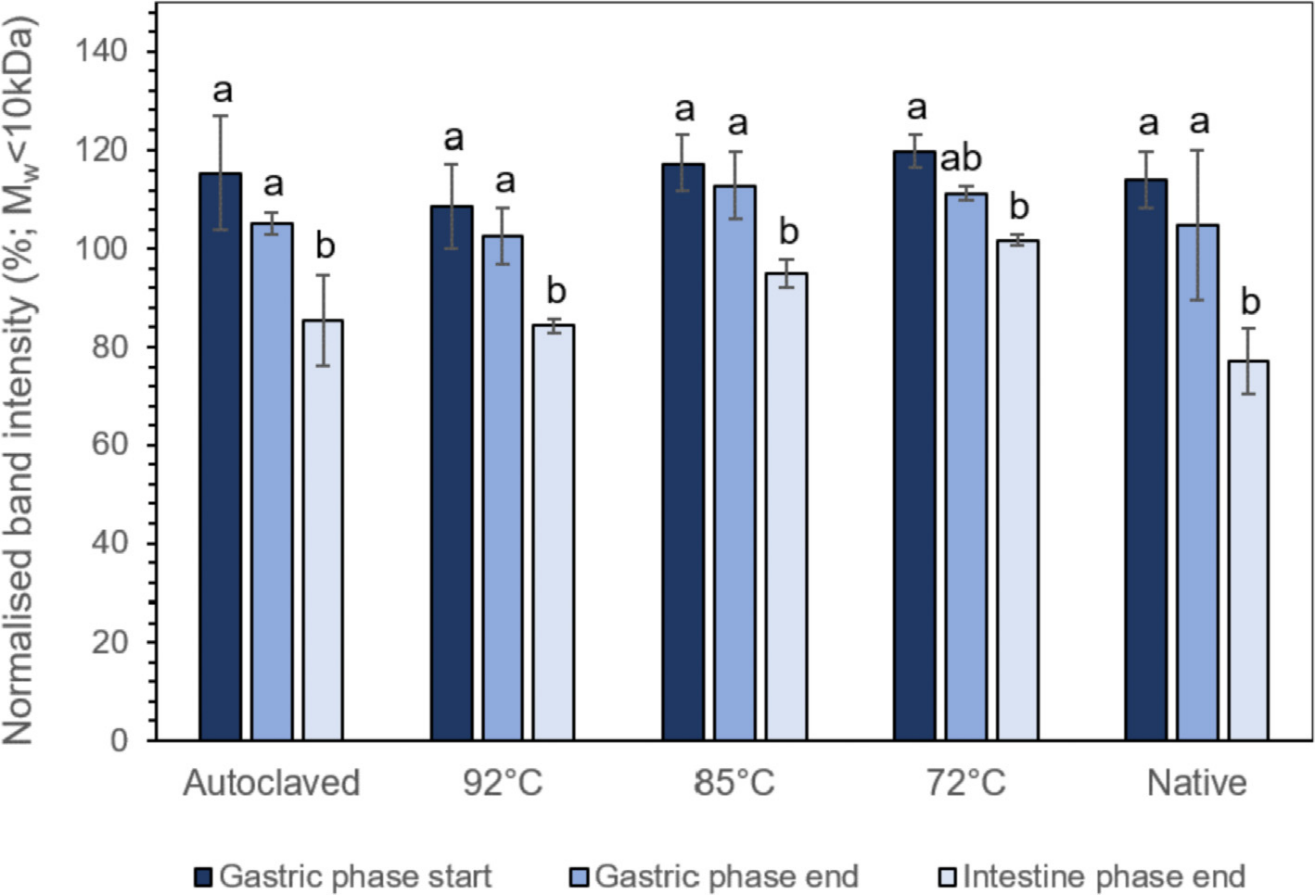
Normalized (to oral phases) intensities of SDS-PAGE protein bands corresponding to a Mw < 10 kDa of the centrifuged gastric (t_GS_ = 0 min and t_GE_= 60 min) and intestinal chymes (t_INT_ = 120 min). ^a-b^ Different letters between the bars indicate significant difference according to Tukey's post hoc means comparison test.

**Fig. 3:**
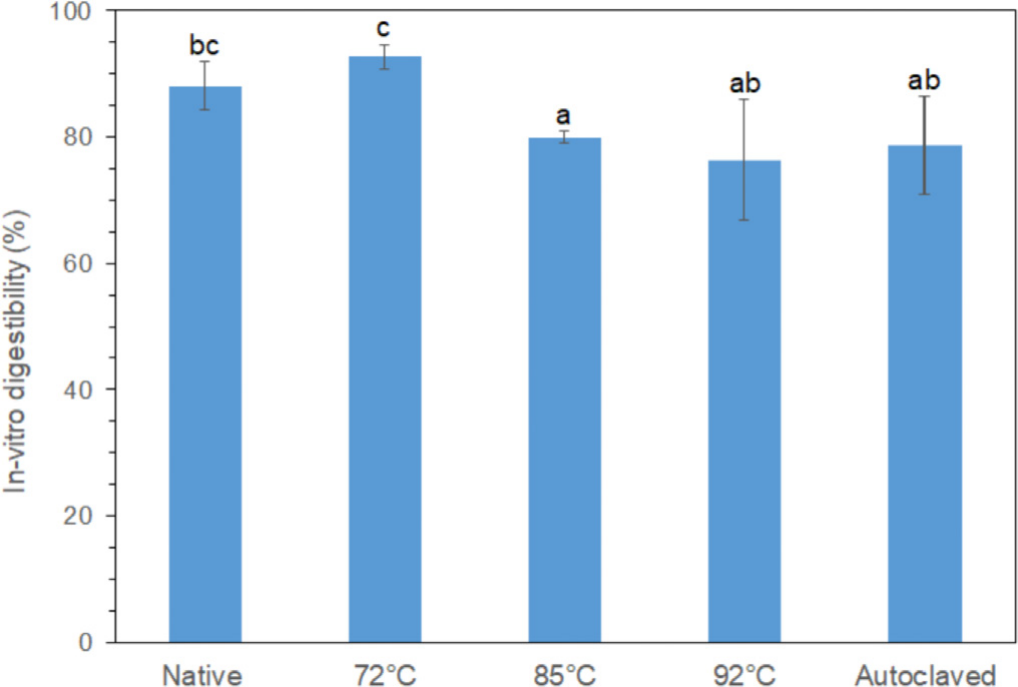
In-vitro digestibility values as determined by means of the soluble nitrogen index between the initial and digested (small intestinal digesta) BSG-PI acid gels systems. ^a-c^ Different letters between the bars indicate significant difference according to Tukey's post hoc means comparison test.

Image analysis of the SDS-PAGE bands corresponding to the average intensities of the three replicates at Mw < 10 kDa, which raw and analyzed data are available in the sheet “<10 kDa signal intensity” of the annexed file “digestibility”, revealed a progressive reduction in the normalized intensities throughout the gastric and intestinal phases (Fig. 2). Interestingly, the in-vitro simulating intragastric processing step did not confer any significant (less than 10%, *p* < 0.05) reduction in the intensity of the Mw < 10 kDa bands. Following 2 h of intestinal digestion, a remarkable decrease in the Mw < 10 kDa bands intensity was observed in all gel systems, indicating that the identified proteinaceous material in the centrifuged digesta was primarily composed of oligopeptides and free amino acids. Indeed, at the end of the digestive process, the in-vitro digestibility ranged from 76 to 93%, the latter corresponding to the acid gels prepared by BSG-PI dispersions heated at 72 °C for 20 min (Fig. 3; raw data and calculation in the file “digestibility”, sheet “In-vitro digestibility”). The high in-vitro digestibility obtained, may be ascribed either to the complex protein composition of the BSG-PIs or to the extensive degradation of the protein material during the malting and mashing process resulting to a broad size distribution of proteins [4].

The aminoacid composition of the native BSG-PI powder are provided as raw data in the file “AA composition.xlsx” available online, including the composition of the 3 amino acid standard mixes used for creation of the standard curve and the resulting quantification of BSG-PI amino acids. The analyzed data expressed on a dry basis of the BSG-PI total solids and protein matter, respectively are presented in Table 1. It was not possible to achieve a satisfactory quantification of methionine, cysteine, and tryptophan due to their partial decomposition during the acid hydrolysis step. Nevertheless, the sum of the three essential aminoacid calculated by difference was estimated at 11.9 g.100 g ^−^ ^1^ of protein matter. In addition, asparagine and glutamine were deamined and converted into aspartic and glutamic acid during the acid hydrolysis, respectively and thus, only one concentration was reported for both aminoacids.

**Table 1:** Amino acid composition of BSG-PI.

Amino acid	g.100 g^−1^ of BSG-PI (dry basis)	g.100 g^−1^ of protein matter (dry basis)	WHO/FAO/UN recommendations^b^ (g)
Cysteine^a^	nd	nd	0.287
Histidine	1.00 ± 0.07	1.33 ± 0.09	0.7
Isoleucine	2.55 ± 0.40	3.39 ± 0.53	1.4
Leucine	4.8*6* ± 0.*50*	6.4*6* ± 0.66	2.73
Lysine	1.83 ± 0.29	2.43 ± 0.38	2.1
Methionine^a^	nd	nd	0.728
Threonine	1.82 ± 0.32	2.42 ± 0.43	1.05
Tryptophan^a^	nd	nd	0.28
Valine	3.48 ± 0.46	4.63 ± 0.61	1.82
Essential	24.47	32.54	11.10

Alanine	3.05 ± 0.37	4.06 ± 0.50	
Arginine	2.60 ± 0.47	3.45 ± 0.63	
Asparagine & aspartic acid	5.21 ± 0.69	6.93 ± 0.91	
Glutamine & glutamic acid	17.21 ± 2.3	22.89 ± 3.05	
Glycine	2.90 ± 0.46	3.86 ± 0.62	
Phenylalanine	4.62 ± 0.49	6.15 ± 0.65	
Proline	9.35 ± 1.19	12.43 ± 1.59	
Serine	3.24 ± 0.35	4.31 ± 0.46	
Tyrosine	2.55 ± 0.41	3.39 ± 0.55	
Non-Essential	50.73	67.46	

a Cysteine, methionine, and tryptophan not determined (nd) due to decomposition during acid hydrolysis;.

b Recommendations for a 70 kg adult per day.

Although the BSG-PI contained a third of essential amino acid, the content was relatively high in the case of the valine (4.6 g.100 g ^−^ ^1^), isoleucine (3.4 g.100 g ^−^ ^1^) and leucine (6.5 g.100 g ^−^ ^1^) considering the FAO recommendations [5] for an adult of 70 kg as reported in the Table 1.

Glutamine and glutamic acid were the most abundant amino acids found in the BSG-PI extract and represented almost one fourth of the amino acids with 22.9 g.100 g ^−^ ^1^. Proline was found to be the second most prevalent amino acid accounting for 12.4 g.100 g ^−^ ^1^ of the total amino acid content. Similar results were reported for pale and black brewers spent grain protein isolates by Connolly et al. (2013) [6].

## 2 Experimental Design, Materials and Methods

1. Acid gel preparation

BSG-PI was extracted under alkaline conditions and acid gel were prepared via δ-gluconolactone (GDL) induced acidification as detailed by Hellebois et al., (2021) [1]. For the preparation of the acid gels, freeze dried BSG-PI were dispersed into MilliQ water (5% wt), adjusted at pH = 7 with NaOH 0.5 M and hydrated for 1 h at 25 °C. Different thermal treatments (i.e. 72, 85 or 92 for 20 min, or autoclaving at 121 °C, 20 min, 1 bar) of the BSG dispersions were conducted. Following thermal treatment, a small amount of sodium azide (0.02% wt) was added into the BSG-dispersions as bacteriostatic. Acid protein gels were prepared by spiking the protein aliquots with 1.2% wt of GDL to commence the *in-situ* acidification. The amount of GDL was calculated to ensure the completion of the hydrolysis of GDL approximately at pH_end_ = 4.2. In the end of the acid gelation process, the gels were broken down using a mechanical stirrer and kept at 4 °C until further use.

2. In-vitro digestion of the protein hydrogels

To monitor their in-vitro digestibility, the acid gels were subjected to in-vitro digestion (in triplicate) implementing the static digestion model (INFOGEST 1.0) as described by Minekus et al. (2014) [7]. In brief, 5 g of stirred acid gel were transferred into a 25 mL glass flask with a stopper and blended with 5 mL with simulated salivary fluid pre-tempered at 37 °C and incubated in a water bath at 37 °C for 2 min. Then, the oral bolus was mixed with simulated gastric fluid, adjusted at pH = 2.5 using HCl 1 M and the obtained gastric chyme was incubated for 60 min under constant shearing at 100 rpm to mimic antral forces in the stomach. Following 1 h of simulated gastric processing, the gastric chymes were blended with the simulated small intestine fluids, adjusted at pH = 7 using NaOH 1 M and incubated under the same conditions for 2 h. At each step of the in-vitro digestion process (i.e. oral phase, initial and end gastric phase, end intestinal phase), aliquots (1 mL) were transferred into Eppendorf tubes, mixed with a proteases inhibitor cocktail (Sigma Aldrich, Leuven, Belgium), quenched with liquid nitrogen and stored at −80 °C until further analysis.

3. SDS-PAGE analysis of the digesta

Protein molecular weight distribution was characterized by reducing SDS-PAGE using a Bio-Rad Criterion™ XT Bis-Tris 12% gel and Precision Plus unstained Protein standards. Samples (8.4 µL) were dissolved in 3 µL of XT Sample Buffer, mixed with 0.6 µL of XT reducing agent, heated at 95 °C for 5 min, loaded (12 µg protein/lane) onto the precast gel and subjected to electrophoresis at a constant voltage of 200 V until the migration front reached the bottom of the gel (45–50 min). After staining with Serva Purple, following manufacturer's instructions, the gel was imaged using a Typhoon FLA 9500 Imager and analyzed using the Image J software. The raw images of the three replicates (SDS-Page Gel 1–3) and the gastric fluids (SDS-PAGE Gel 4) were uploaded to the linked repository. The information about the loadings in each well of the 4 gels are reported in the file “digestibility” sheet “SDS-PAGE Lanes information” of the annexed documents.

4. In-vitro digestibility determination

The in-vitro digestibility of the protein acid gels was measured in duplicate via the total nitrogen quantification method as described in Hu et al. (2019) [8]. Briefly, 5 mL of the intestinal chymes were centrifuged at 8000 *g* at 4 °C for 20 min. The soluble nitrogen content of digesta supernatant was determined by the Kjeldahl method and the in-vitro digestibility was expressed as follows:

$$\text{In}\text{-}\text{vitro}\;\text{digestibility}\;=\;\frac{\text{Protein}\;\text{content}\;\text{in}\;\text{the}\;\text{digesta}\;\text{supernatant}\;\times \;\text{dilution}\;\text{factor}}{\text{Protein}\;\text{content}\;\text{of}\;\text{the}\;\text{initial}\;\text{acid}\;\text{gel}}$$

Raw data of the results obtained for two replicates of the gels for the five condition are presented in the excel file “digestibility” sheet “in-vitro digestibility”.

5. Amino acid quantification

Prior to the quantification of the amino acid, a complete hydrolysis of the protein was made. Briefly, a small amount (ca. 5 mg) of BSG-PI was added to an empty Pyrex tube in 4 replicates and 1 mL of HCl 6 M was added. The protein isolates were hydrolysed for 24 h at 110 °C in an oven (Hach, Loveland, United States). The solution was then diluted to 10 mL using deionized water and filtered through a 0.2 µm PFTE filter. The obtained samples were then diluted to 3 concentrations (approx. 0.5, 0.125 and 0.05 g.100 mL^−^^1^) to be within the standard curve range. Three standard sample mixes with different amino acid composition as detailed in the linked document stored in the repository (“AA composition”) were prepared at 0.02, 0.04, 0.06 and 0.08 mM to build the standard curve for each amino acid.

Qualitative and quantitative analysis of amino acids was realized on a HPLC-MS system (ThermoFisher Scientific, San Jose, CA, USA) consisting in a binary solvent delivery pump connected to a photodiode array detector (PDA) and a LTQXL mass spectrometer equipped with an atmospheric pressure ionization interface operating in electrospray positive mode (ESI+). Ten microliters of extracts were separated on a C18 column (150 × 2.1 mm, 5 µm) (Alltima, Hichrom). The flow rate was set at 0.2 mL.min^−1^ and mobile phases consisted in water modified with NFPA (20 mM) for A and pure acetonitrile for B. Amino acids were eluted using a linear gradient from 7 to 25% of B for 12 min and then an isocratic step at 25% of B during 18 min. Mass spectrometric conditions were as follows: spray voltage was set at 4.5 kV; source gases were set (in arbitrary units min^−1^) for sheath gas, auxiliary gas and sweep gas at 30, 10 and 5, respectively; capillary temperature was set at 250 °C; capillary voltage at 5 V; tube lens, split lens and front lens voltages at 30 V, −62 V and −8.75 V, respectively. Ion optics parameters were optimized by automatic tuning using a standard solution of arginine at 0.1 g.L ^−^ ^1^ infused in mobile phase (A/B: 50/50) at a flow rate of 5 µL.min^−^^1^. Full scan MS spectra (70 to 210 m/z) were performed on a LTQ analyzer (Linear Trap Quadripole). Raw data were processed using the XCALIBUR software program (version 2.1, http://www.thermoscientific.com). The result obtained presents the average of at least the four replicates of the acid hydrolysis. When two concentrations (i.e. 0.5, 0.125 or 0.05 g.L ^−^ ^1^) were within the standard curve, both results were used to quantify the amino acid. In addition, the results for the same amino acid from different standard mixes were averaged. All results had the same weight in the quantification.

6. Statistical analyzes

The significance of the thermal process on the in-vitro digestibility and intensity of the SDS-PAGE molecular weight bands (< 10 kDa) was determined by means of one-way Analysis of Variance (ANOVA). Tukey's multiple range test was used to separate means of data when significant differences (*p* < 0.05) were found. All univariate statistics were performed using SPSS software (IBM, France).

## Ethics Statement

This article conforms to Elsevier's standards of ethical publishing.

## CRediT Author Statement

**Thierry Hellebois:** Conceptualisation; Investigation; Formal Analysis; Writing – original draft; **Claire Gaiani:** Conceptualisation; Supervision; Writing – review & editing; **Cédric Paris:** Investigation; Writing – review & editing; **Sébastien Planchon:** Investigation; Writing – review & editing; **Jenny Renaut:** Writing – review & editing; Project administration; **Christos Soukoulis:** Conceptualisation; Supervision; Project administration; Writing – review & editing.

## Declaration of Competing Interest

The authors declare that they have no known competing financial interests or personal relationships, which have, or could be perceived to have, influenced the work reported in this article.

## Appendix Supplementary materials

Image, application 1
